# Language-dependency of the left-digit effect in number line estimation and the role of number word inversion

**DOI:** 10.1038/s41598-026-54579-w

**Published:** 2026-05-27

**Authors:** Elena Sixtus, Jan Lonnemann, Song Yan, Peng Li, Silke M. Göbel

**Affiliations:** 1 Empirical Childhood Research, University of Potsdam, Karl-Liebknecht-Str. 24-25, 14476 Potsdam, Germany; 2 Constructor University, Bremen, Germany; 3 Yunnan Normal University, Kunming, China; 4 University of York, York, UK; 5 Centre for Research in Equality in Education (CREATE), University of Oslo, Oslo, Norway

**Keywords:** Left-digit effect, Inversion effect, Cross-cultural comparison, English, German, Mandarin, Neuroscience, Psychology, Psychology

## Abstract

Language plays a critical role in shaping cognitive processes. The present study focuses on the impact of language-dependent number representations, specifically examining the effects of number word inversion and the left-digit effect across different linguistic groups. Number word inversion is a phenomenon where in certain languages, such as in German, spoken two-digit numbers start with the unit preceding the decade (e.g., for 91, literally “one-and-ninety” rather than the Arabic numeral order “ninety-one”). Previous studies employing various numerical tasks indicate that the inversion effect—where the unit digit exerts a disproportionate impact on how the total magnitude of a two-digit number is perceived—influences children’s performance. This study addresses the inversion effect in adults by comparing German-speakers to English- and Mandarin-speakers in a number line estimation task. Linear regression slope analyses of estimated positions conducted across decades identified the inversion effect, demonstrating that German-speakers provided comparatively higher estimates for numbers with larger unit digits compared to the other language groups. Additionally, the left-digit effect, which conversely is characterised by the smaller impact of the unit digit on perceived number magnitudes, became evident in all three language groups. This left-digit effect is further modulated by membership of a specific language group. The structure of Mandarin number words is discussed as a significant factor contributing to the pronounced amplification of the effect. Our findings suggest that language plays a pivotal role in persistently shaping the cognitive processing of numbers.

## Introduction

Numbers are omnipresent in virtually everybody’s life and numeracy skills have been shown to be predictive of people’s economic success throughout life^1,2^. Accordingly, a large body of research addresses the questions of how humans understand and represent numbers, and how they acquire numerical knowledge in the first place. A multitude of research methods is used to gain insights into numerical development and mental number representations. The number line estimation (NLE) task is often used for several reasons. Firstly, it is very easy to implement. Usually, numbers are presented visually or auditorily and participants have to mark on a line where they think the numbers should go. Endpoints of the line are either marked, e.g., 0 on the left side and 100 on the right side, or not marked. Assuming a linear distribution of numbers along the line, each number has a theoretical ‘true’ position. Participants’ number placing positions or estimation errors can then be used for further analyses. Secondly, the number line is often thought to play a special role in number representations. At least one well recognised model of number processing claims that the mental representation of non-symbolic number size is based on its representation along a mental number line^3^. This mental number line consists of small numbers on the left side and consecutive larger numbers towards the right side (for Western cultures^4^). Estimation errors in a NLE task should therefore specifically measure the accuracy of mental number representations. Based on this assumption, models for the development of mathematical competencies assume number representations along a number line as a necessary step on the way towards a mature number representation^5,6^. Thirdly, better performance in NLE tasks has been shown to be associated with higher mathematical proficiency^7–10^. Therefore, whether or not NLE tasks measure the ‘true’ number representation, their outcome is relevant regarding real-world mathematical outcomes. 

### Sources for systematic estimation errors in the NLE task

The NLE task usually requires the translation of a number word or an Arabic number symbol into a position on a line. Therefore, specific characteristics of the original format may play a role in this task. For example, in some languages number words are inverted. That is, in spoken two-digit numbers the unit precedes the decade, although in written Arabic numbers the decade precedes the unit (e.g., in German 91 is “ein-und-neunzig”: “one-and-ninety” instead of “ninety-one” in English which follows the written order of decade and unit). This inversion property evokes specific errors in numerical tasks. For example, Moeller and colleagues^11^ found more frequent transcoding errors, and specifically more inversion errors in a writing-to-dictation task for German (a language with number word inversion) than Japanese (a language without number word inversion) speaking 6- to 7-year-olds. Increased transcoding costs were also observed among Arabic- and Hebrew-speaking adults — that is, in cultures with a right-to-left reading direction — with both groups being slower at transcoding three-digit numbers with inverted presentation order of unit-decade^12^. Note that while this inverted order is the standard order in the Arabic language, Arabic-speaking individuals were nonetheless also faster when presented with the non-standard, but non-inverted decade-unit order^12^. The inversion property of one’s native language also appears to impair memory performance when recalling lists of two-digit numbers^13^. The language-specific influence of the inversion property has furthermore been shown in children’s reading and writing^14^, arithmetic^15^, and magnitude comparison^16^ performance. An instance of the inversion effect in the context of the NLE task is described by the finding that in “inverting” languages like German estimation errors are bigger than in a language without inversion property. Specifically, Helmreich and colleagues^17^ tested Italian-speaking and Austrian (German-speaking) children with a pen-and-paper NLE task. Each child received 18 one- and two-digit target numbers, of which eight were relevant for the research question regarding the role of number word inversion. German-speaking children’s estimation error was on average higher than those of Italian-speaking children, in particular for numbers with large decade-unit-distances (e.g., 27 has a large decade-unit-distance of *7 − 2 = 5*, while 23 has a small decade-unit distance of *3 − 2 = 1*). The authors mainly attribute this result pattern to German-speaking children mixing up the decade and unit digit. Another factor that could drive such result patterns is the emphasis on the unit digit in inverting languages. Consequently, it is conceivable that the overrepresentation of the unit digit, or a categorical misconception of inverted number words manifests in these numbers’ mental number size representations to be observable still in adults. Savelkouls and colleagues^18^ provided evidence for this, showing that adult Dutch-English bilinguals (with mostly Dutch, a language with inverted number words, as their first native language) did not display a left-digit effect. The left-digit effect, in contrast to the inversion effect, indicates an overrepresentation of the leftmost digit, which in two-digit numbers is the decade digit. It therefore concerns another specific characteristic of the original number format - here: visual presentation of Arabic target numbers - that may play a role in NLE tasks. In the study by Savelkouls et al.^18^, participants also performed a NLE task. Monolingual English participants’, but not the bilinguals’, response patterns reflected that they systematically overestimated the distances between numbers around a decade break (e.g., 29/31 was placed more than 2 units apart). In other studies, the left-digit bias was analysed using a modeling approach. Patalano and colleagues^19^ fitted models reflecting the general response tendencies in NLE of test subjects. The contribution of the unit digits to the placement positions was then determined by a newly introduced parameter. Results showed that two-digits’ unit digits contributed less to participants’ placing positions in the NLE task, conversely showing the larger contribution of the decade that is the left digit. 

#### The role of language in mental number representations

Earlier, we briefly touched on the possible influence of the structure of number words on the mental representation of numerical magnitude. This could result from the regular use of essentially misleading number words, whereby numbers become associated with magnitudes that differ from those ‘objectively’ being referred to. This presupposes the widely accepted automatic co-activation of magnitude representations when using number symbols (e.g., number words or digits)^3,20^. In other words, linguistic features could ultimately determine, in part, an individual’s perception of the underlying non-linguistic concepts. Other researchers reached a similar conclusion resembling the Whorf hypothesis^21^, or a weak hypothesis of linguistic relativity. For example, Pixner and colleagues^22^ found a stronger unit-decade compatibility effect in German-speaking children than in speakers of two languages without a consistent number word inversion property (Italian and Czech) in a magnitude comparison task with Arabic two-digit numbers.

The mechanism behind the distortion of the mental representation of number size might be that an emphasis on the first part of verbal number words leads to its mental overrepresentation. Following this argument, languages with number word inversion (e.g., German, Dutch, Arabic) should lead to under-/overestimation of numbers with relatively small/large unit digits, respectively, whereas speakers of languages without number word inversion might tend to neglect the (secondly spoken) unit digit, leading to increasing underestimations of numbers with increasing unit digits, and thus a leap to the next decade. A language used in many studies as an instance of a non-inverting language is English. Interestingly, in English, teen numbers are actually inverted (e.g., “fourteen” instead of “ten-four”). While one can argue that all numbers from 20 on can be used as non-inverted number words, English-speakers’ commonplace exposure to number word inversion for part of their number set might influence their general two-digit number processing. This so far very tentative claim could be addressed by a direct comparison with speakers of languages that have a completely consistent decade-unit number word system. One example of such a strictly non-inverting language is Mandarin. Assuming that the structure of the number word system influences individuals’ magnitude representations, one should expect Mandarin-speakers to show an even stronger neglect of the unit digit than English-speakers. 

##### The present study

The present paper examines the influence of native language on mental number representations, in particular it investigates the inversion effect and the left-digit effect. The inversion effect has to date only been tested with relatively few two-digit numbers on a horizontally aligned number line in children. In our experimental design, we followed the basic approach of this earlier study^17^ and used a non-verbal NLE task with visually presented target numbers. Furthermore, we measured spatial representations on different dimensions in English, German, and Chinese adults with a larger number of two-digit numbers in a computerized 0-100 NLE task on horizontally as well as vertically oriented number lines. Our main research question was whether the inversion effect also appears in adults. Thus, the main hypothesis concerned a systematic distortion of NLE as a function of unit digit size specifically of German-speakers because of the German language’s consistent number word inversion. Furthermore, we tested whether number word inversion at the item level is driving the effect, by comparing inverted and non-inverted number words within one language. Because in English, only teen-number words are inverted, we hypothesized that teen-numbers would be overestimated in direct comparison with twenty-numbers, whose corresponding number words are not inverted. The study was preregistered under https://osf.io/yjqbv for the English- and German-speaking sample, but we finally also had the opportunity to test Mandarin-speakers, and improved analyses that were not preregistered also included the left-digit effect and its modulation by native language. The addition of the Mandarin-speaking sample enabled us to furthermore compare the German, strictly inverting number word system and the English, inconsistently non-inverting system, with an entirely non-inverting and highly regular number word system. Our main expectation was that Mandarin-speakers, in contrast to German- and English-speakers, would not exhibit an overestimation of teen-numbers in relation to twenty-numbers.

## Methods

### Participants

In England, 85 English-speaking, and in Germany 81 German-speaking subjects completed the study in return for course credit or payment. Of the English sample, four participants were excluded from further analyses because they had at least one second native language with number word inversion property (German, Nepali and Hindi, Urdu, Dutch). Apart from that, one English-speaker listed French as an additional native language and one German-speaker listed Kurdish. Their data remained in the data set to be analyzed. Furthermore, three German- and one English-speaking participants were excluded because of response patterns that revealed a lack of comprehension of the task or an unwillingness to complete it properly: one English- and two German-speaking participants always touched the line more or less at the midpoint, possibly indicating a misunderstanding that they should touch the line where they had previously seen the number, and one German-speaking participant switched between a left-to-right/right-to-left, and a bottom-to-top/top-to-bottom oriented number line, and also violated task instructions regarding an unchanging response hand (noted by the experimenter). This left data of 80 English- and 78 German-speaking participants. The English-speaking sample exceeds the preregistered number by two, because participant registrations continued before it was certain that sufficient complete data sets were obtained. In addition to the preregistered English- and German-speaking sample, we were able to collect further data from a Chinese, Mandarin-speaking sample. Here, 78 participants took part in the experiment, of which two data sets were excluded because they also switched between a left-to-right/right-to-left oriented number line, leaving 76 complete Chinese data sets. Of the final sample, mean age was 21.88 years (*SD* = 5.80, English-speakers: *M* = 21.42, *SD* = 5.71, German-speakers: *M* = 24.58, *SD* = 7.33), Mandarin-speakers: *M* = 19.59, *SD* = 1.37). There were 209 right-handers (of which 71 were English-speaking, 64 German-speaking, and 74 Mandarin-speaking), 15 left-handers (8 English-speaking, 5 German-speaking, and 2 Mandarin-speaking), one English-speaking was ambidextrous and nine German-speakers whose handedness data was not recorded. There were 160 female (62 English-speaking, 57 German-speaking, and 41 Mandarin-speaking), 63 male participants (17 English-speaking, 11 German-speaking, and 35 Mandarin-speaking), one English and one German participant identified with non-binary gender and gender data from nine German-speakers was not recorded. The study was approved by the Ethics Committee of the University of York, the Ethics Committee of the University of Potsdam, and the Faculty of Education’s Ethics Committee at Yunnan Normal University. All methods were performed in accordance with the relevant guidelines and regulations, including the Declaration of Helsinki. All subjects gave written informed consent.

### Apparatus

Participants were individually tested. They were seated at a table facing a tablet PC (Microsoft Surface Pro 3) that was set up in horizontal or vertical orientation. The display scale was set to 150% as standard. Around the display, black tape was stuck over the edges (including the camera and Home icon) of the tablet. In front of the tablet, there was a centrally positioned keyboard with the central “-“-key (English layout) marked by a yellow sticker. Visual stimuli were presented on the display (60 Hz refresh rate, 12” screen diagonal). The experiment was controlled and data recorded by expyriment software^23^. Participants were seated so that they could reach the marked key and the tablet comfortably, resulting in a viewing distance of about 50 cm from the display.

### Stimuli

Target numbers in both tasks were visually presented Arabic two-digit numbers: 13, 14, 15, 16, 17, 18, 19, 21, 23, 24, 25, 26, 27, 28, 29, 31, 32, 34, 37, 39, 41, 42, 43, 46, 48, 51, 52, 54, 56, 58, 62, 63, 65, 67, 69, 72, 73, 76, 78, 79, 82, 84, 85, 87, 89, 91, 92, 93, 96, and 98. Target numbers for training trials were 70, 30, 50. Font size in all cases was 512 pixels, sans serif font type, white on black background. The number line was presented as a straight, white line on black background, ranging from coordinate x=-500, y = 0 to x = 500, y = 0. The line was horizontal or vertical depending on the orientation of the tablet PC. When participants responded by touching the screen, the recorded position was shortly marked by a small green point (radius = 5 pixels).

### Procedure

**Fig. 1:**
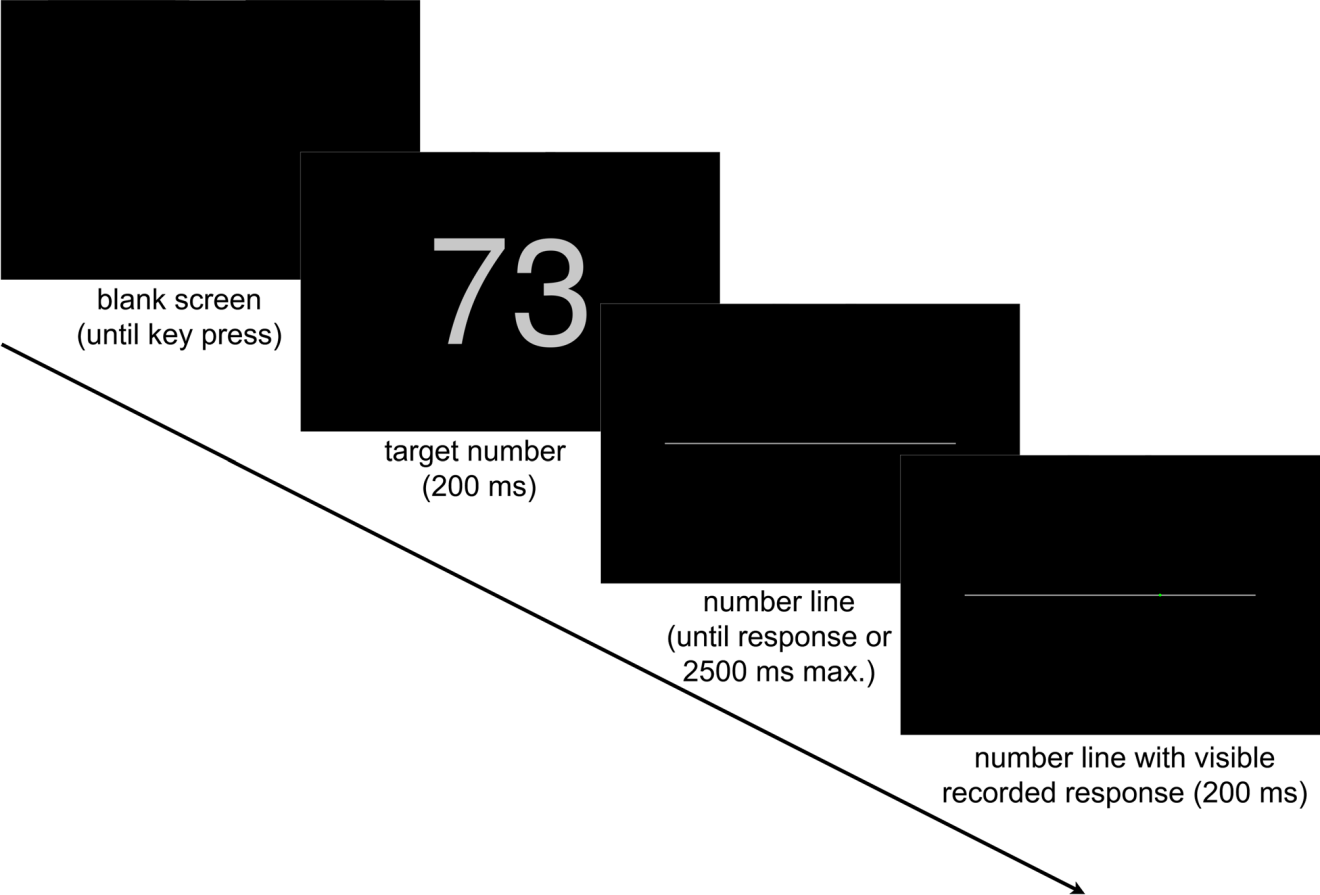
Example of the procedure in the tablet task, horizontal number line.

Participants were instructed to sit centrally in front of the tablet PC and to use their dominant hand both for pressing the yellow marked key to initiate each trial and for touching the tablet display at the point where they estimate the visually presented number should go. They were informed that numbers would range from 0 to 100, but not at which end of the line each of these numbers should be. When the participant pressed the yellow marked key, the target number immediately appeared on the display and was replaced by the number line after 200 ms (see Fig. 1). Response time measurement started with line presentation. If participants failed to respond within 2500 ms, the trial terminated, that is, the line disappeared and the next trial was to be initiated by key press. The missed trial was then automatically repeated at the end of the experimental block. After participants’ response, a green dot marked the touch position for 200 ms until the screen went all black to indicate that the next trial could be initiated.

Instructions explicitly stated that participants should not read the numbers aloud, should always act intuitively and as fast as possible, and should try to really estimate the position without applying any other strategy.

After the main task, a short questionnaire on the tablet PC inquired about the participant’s age, gender, handedness, and native language(s).

### Design

Each target number was presented four times per line orientation. Thus, the experiment comprised 400 trials: 50 numbers x 4 repetitions x 2 orientations. Number order was quasi-randomized: the randomization algorithm at the start of each run ensured that consecutive numbers did not have the same unit and they had a numerical distance of at least 10. This rule could potentially only be violated in case of previously missed (timed-out) trials that were repeated at the end of the experimental block. Half of the participants started with the horizontal tablet orientation and the other half started with the vertical tablet orientation. In the beginning of the task and after changing the orientation, a training took place. The training comprised three trials with target numbers 70, 30, and 50 (in this order). A training trial was repeated at the end of the training (as was an experimental trial in the end of the respective experimental block) in the case of a missed response (time-out after 2500 ms). The whole experiment (excluding the “floor task”, see below) took about 20–30 min.

### Floor task

For a subsample of the English- and German-speaking participants, the task was furthermore implemented in an adapted version (“floor task”) on a bigger scale on the floor with tape denoting the (lateral and sagittal) line and objects to be placed on the line (instead of positions to be touched on the tablet) which were then measured by time of flight sensors at the ends of the line. The floor task was added on an exploratory basis as an extension of the horizontal and vertical axes of the tablet task to include the lateral and sagittal axes. It was administered to as many participants as possible, but for logistical and scheduling reasons not to all of them. The whole experiment including the floor task took about 45–60 min. All participants started with the tablet task. Further information, and in particular analyses regarding the floor task are presented in the supplementary materials only, mainly because they did not substantially add to the analyses from the tablet task.

### Data analysis

We used R (Version 4.5.1^24^) and the R-package *papaja* (Version 0.1.3^25^) for all our analyses. Responses were given in the form of touching a line presented on a tablet PC. The line represented the number line and was presented in the horizontal and vertical orientation. As participants were not informed about the “small” and “large” end of the number line, their choices varied, mostly in the vertical orientation, but also for a few in the horizontal orientation. The chosen line orientations are reported in Table 1.

**Table 1:** Number of used line orientations per task and per mother-tongue.

Mother-tongue	Left-to-right	Right-to-left	Bottom-to-top	Top-to-bottom
English	79	1	65	15
German	77	1	65	13
Mandarin	75	1	52	24

As described in the preregistration, responses were excluded if the x-coordinate of the response lay beyond the presented line or if the y-coordinate was more than 150 pixels away from the y-coordinate of the line. This affected a total of 68 responses (0.07% of all responses). For all analyses, touch positions were then converted to a uniform scale: pixels from the tablet PC were converted to units corresponding to the scale of the target numbers and the right-to-left and top-to-bottom number lines were numerically “turned around” so that always, a smaller outcome corresponds to a response towards the “smaller end” of the number line, (e.g., outcome *25* corresponds to a touch position at 1/4 of the notional 0-100 line, irrespective of line orientation). This outcome, i.e., the converted measured positions, will hereafter be called *placing position*.

### Overview over general response patterns

First of all, for an overview over general response patterns, we report effects of participants’ mother-tongue and of the number line’s orientation on positioning bias and on absolute positioning bias. “Positioning bias” is the difference between the placing position (x-coordinate, i.e., along the printed line) and the “true” position of the respective target number assuming a linear, equidistant distribution of numbers 0-100 on the line. This value also reflects general under-/overestimation biases. “Absolute positioning bias” is the same, only that all values are converted to absolute values first, so that positive and negative values do not cancel each other out. It is equivalent to “percent absolute error” (PAE) used in many other studies. We conduct two mixed ANOVAs, both with the within-subject factor Dimension (horizontal/vertical) and the between-subject factor Mother-tongue (English/German/Mandarin) first on the dependent variable Positioning bias, then on Absolute positioning bias.

### Response times

To gain a further first overview of response behaviour, we show the response time (RT) data in comparison with the absolute positioning deviations in a purely descriptive manner. Here, it is possible to discern whether there is a speed-accuracy trade-off or whether the response time data mirror the positioning behaviour. A speed-accuracy trade-of would be indicated by longer RTs for smaller biases and vice versa.

### Preregistered analyses

In the preregistration (see https://osf.io/yjqbv), we formulated two main hypotheses. As elaborated further below, these turned out to be problematic, therefore we added improved analyses that were not preregistered.

(1) Between languages: The stimulus set includes pairs of inverted two-digit numbers (e.g., 23–32, 37–73, …). In German-speakers (but not or less in English-speakers) we expected overestimations of numbers with larger units than decades (e.g., 28) and underestimations of numbers with smaller units than decades (e.g., 82). We therefore expected the average distances between placing positions for each of these number pairs to be smaller for German- than for English-speakers. This was to be realized by a one-tailed independent *t*-test with the independent variable Native language (English/German), and the dependent variable Distance between placing positions of inverted number pairs. Only target number pairs with both numbers above 20 were used here. (Specifically, number pairs were: 23–32, 24–42, 25–52, 26–62, 27–72, 28–82, 29–92, 34–43, 37–73, 39–93, 48–84, 56–65, 58–85, 67–76, 69–96, 78–87, 89–98.)

(2) Within English-speakers: In contrast to number words for numbers larger 20, most English teen-numbers are inverted. Therefore, we expected native English-speakers to overestimate teen-numbers (13–19). This is tested by comparing the positioning bias of those numbers against the positioning bias of the numbers with the same units of the closest decade (numbers 23–29) and we expected a stronger overestimation for teen- than twenty-numbers. This was to be realized by a one-tailed paired *t*-test with the independent variable Decade (Teens: 13–19/Twenties: 23–29), and the dependent variable Positioning bias.

### Evaluation of preregistered analyses and description of further analyses

Regarding the first hypothesis, the most relevant number pairs, being the ones with large decade-unit distances, are at distant sides of the number line (e.g., 29–92). This leads to the problem that any tendency to (de)compress the number line, that is, generally place numbers more or less towards the ends of the line, systematically influences the distance between the number pairs’ positions on the line. The most problematic confounding factor might be that numbers with larger unit digits than teen digits tend to be on the “small side” of the number line, and vice versa with numbers with smaller unit digits than teen digits on the “large side” of the number line. In fact, to examine the language-comparative inversion effect, it is indeed preferable to conduct an analysis that focuses on the influence of exclusively the unit position. Ideally, it should furthermore take all target numbers into account. We therefore examined the units within each decade: German-speakers should be more affected by the size of the unit digit than English-speaking participants due to the structure of the number words. German-speakers should therefore reveal steeper (positive) slopes over units (within a decade) than English-speaking participants (excluding the first decade because teen numbers are also inverted in English). Importantly, the preregistered analyses did not mention the examination of data from Mandarin speakers, as the opportunity to collect these only arose later. Naturally, further analyses will include these additional data. Not featuring number word inversion, Mandarin speakers should also reveal flatter slopes than German-speakers. We therefore conducted two one-tailed *t*-tests comparing German vs. English-speakers’ and German vs. Mandarin speakers’ mean individual linear regression slopes. More specifically, placing positions were regressed on target numbers, individually for each participant and each decade (> 20). The extracted values for slopes were then used as the dependent variable. We expected an inversion effect as indicated by significantly larger average slopes for German-speakers than for English-speakers and for Mandarin speakers. For the sake of completeness, a two-tailed *t*-test furthermore compared English and Mandarin speakers’ average slopes.

Regarding the second hypothesis, we had disregarded the general tendency to underestimate numbers’ positions. We therefore additionally conducted the analyses on the data of the other language groups to be able to compare response patterns between languages and thereby to put the results into perspective.

### The left-digit effect

Another way to look at the inversion effect is to frame it as (the reduction of) a left-digit effect. Very recently, models were proposed and refined to illustrate the general left-digit effect^19^. In summary, the authors first modeled general response tendencies in NLE of test subjects (in the form of one- and two-cycle models) and then introduced a variable $\:\delta\:$ allowing to reflect the lesser contribution of the unit digit in two-digit numbers to the placement position. The authors reported two improved models, the “modified” power model, reducing only the unit digits’ contribution, and the “expanded” power model, reducing both the unit digit’s and the decade digits’ contribution. As we were specifically interested in the language-specific unit digits’ influence on number representations, we adopted the “modified” power model, introducing a further variable $\:\lambda\:$, allowing each language its own $\:\delta\:$ value. Patalano et al.^19^ further distinguished between a one-cycle and a two-cycle model that differ in their overall curvature, as only the two-cycle model assumes the midpoint as an additional reference point. They assigned each subject the model that best matched their response pattern, because their focus was on further improving the model. However, our focus is on determining the language-specific influence of the unit digit, which is why the basic model should remain the same for every subject. We chose the two-cycle model for this purpose, as it better represented the data overall in terms of a lower Bayesian Information Criterion, or BIC, score. However, using the one-cycle model instead of the two-cycle model yields the same result patterns.

#### The modified two-cycle power model and the introduction of the language modifier

The modified two-cycle power model, developed by Patalano and colleagues^19^, captures the global cyclical pattern and a left-digit bias. The model’s equation is shown first below, where y = predicted target placement, $\:{x}_{d}$ = decade of target value (e.g., *20* in 28), $\:{x}_{u}$ = unit digit of target value (e.g., *8* in 28), $\:\beta\:$ = index of curvature, $\:\delta\:$ = (power) weight of unit digits, UB = upper boundary, and LB = lower boundary, with these boundaries depending on whether numbers are below versus above the midpoint of the target range/line. The parameters’ starting values are $\:\beta\:$ = 1, $\:\delta\:$ = 1.

$$\:y=\frac{{\left(\left({x}_{d}+{x}_{u}^{\delta\:}\right)-LB\right)}^{\beta\:}}{\left({\left(\left({x}_{d}+{x}_{u}^{\delta\:}\right)-LB\right)}^{\beta\:}+{\left(UB-\left({x}_{d}+{x}_{u}^{\delta\:}\right)\right)}^{\beta\:}\right)\mathrm{*}0.5}\mathrm{*}100+LB$$

where $\:\beta\:>0;$ for $\:0\le\:x\le\:50$, LB = 0, UB = 50; for $\:50<x\le\:100$, LB = 50, UB = 100.

The language modifier $\:\lambda\:$ (starting value = 0) is introduced to modify each $\:\delta\:$ value depending on the language group, resulting in the equation:

$$\:y=\frac{{\left(\left({x}_{d}+{x}_{u}^{\left(\delta\:+\lambda\:\mathrm{*}L\right)}\right)-LB\right)}^{\beta\:}}{\left({\left(\left({x}_{d}+{x}_{u}^{\left(\delta\:+\lambda\:\mathrm{*}L\right)}\right)-LB\right)}^{\beta\:}+{\left(UB-\left({x}_{d}+{x}_{u}^{\left(\delta\:+\lambda\:\mathrm{*}L\right)}\right)\right)}^{\beta\:}\right)\mathrm{*}0.5}\mathrm{*}100+LB$$

where $\:\beta\:$, LB, UB as above and L = 0 for reference language, L = 1 for language to compare.

The value of interest here is $\:\lambda\:$. If it proves to be significantly different from 0, this reveals the language-specificity of the unit digits’ contribution to the placement positions. The model will be run three times: {1} to compare English- against German-speakers (i.e., $\:{L}_{English}=0$, $\:{L}_{German}=1$), {2} to compare English against Mandarin speakers (i.e., $\:{L}_{English}=0$, $\:{L}_{Mandarin}=1$), and {3} to compare German against Mandarin speakers (i.e., $\:{L}_{German}=0$, $\:{L}_{Mandarin}=1$). For these analyses (and only here), the median is used instead of the mean to average placing positions, mainly because this is adopted from the original formulation of the model^19^. Furthermore, we chose not to exclude responses on the basis of larger positioning biases (as was done before^19^), but each participant responded to each target number eight times (instead of once), so using the median, we were confident that potential outliers would not distort the estimated average placing positions. 

##### Regarding test corrections, significance level, and statistics software

In all analyses, degrees of freedom are Greenhouse-Geisser or Welch-corrected where necessary. The threshold for statistical significance is assumed at *p* = .05, unless stated otherwise. Following up ANOVAs’ significant main and interaction effects, contrasts from estimated marginal means (using the R package *emmeans*^26^) with Tukey-adjustment of *p*-values for multiple comparisons are reported to identify the source of the effect. For ANOVAs, we use the R package afex^27^, for estimating the model parameters, *nlstools*^28^ is used. Effect sizes for *t*-tests are reported as Cohen’s *d*, from the effect size package^29^.

## Results

### Overview over general response patterns

**Fig. 2:**
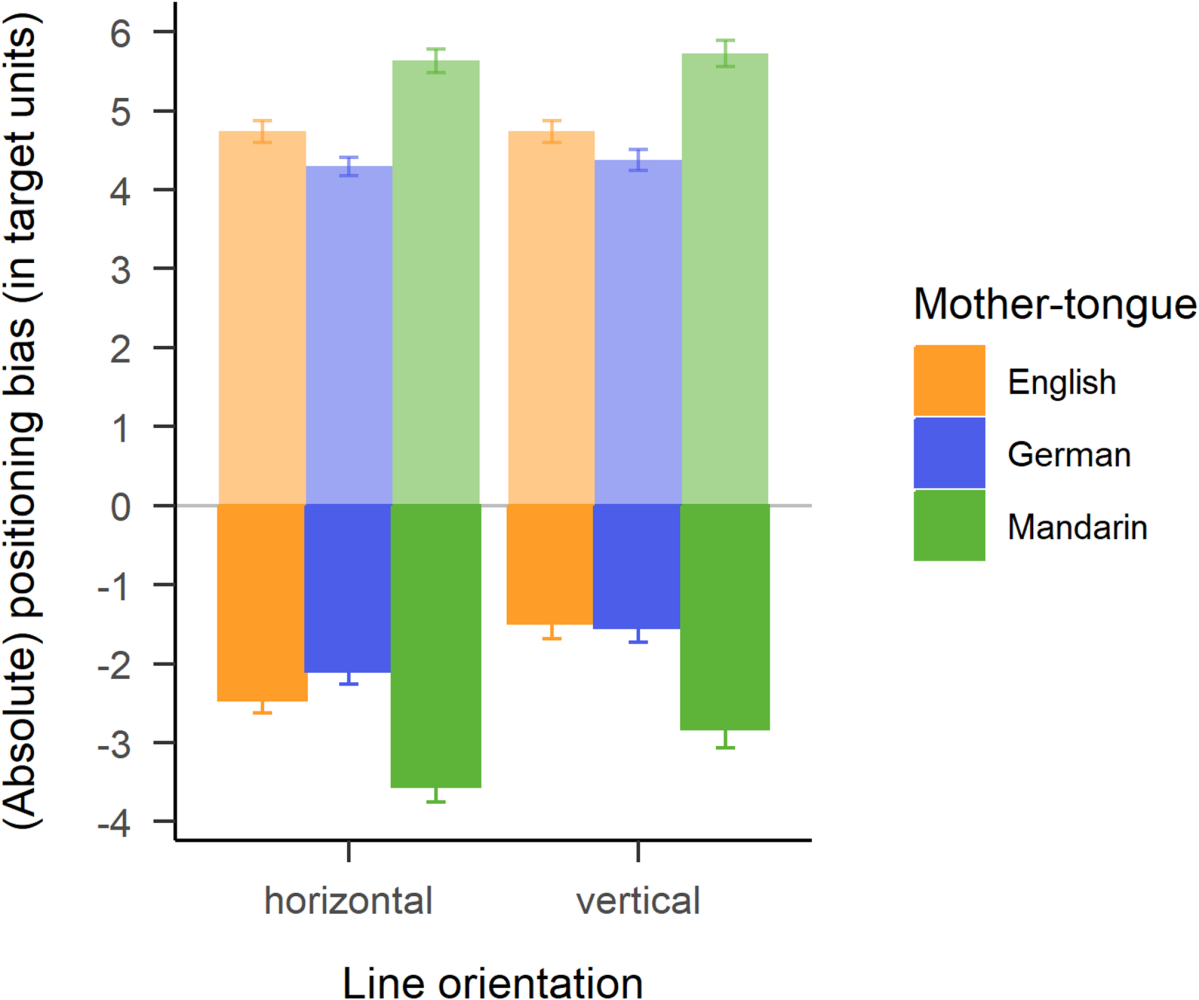
Average absolute positioning bias (transparent bars) and average positioning bias (solid bars) for the three language groups in each line orientation. Error bars display the standard error of the mean.

The mixed ANOVA on Positioning bias yielded significant main effects of Mother-tongue, $\:F\left(2,231\right)=20.42$, $\:p<.001$, $\:{\widehat{\eta\:}}_{G}^{2}=.113$, 90% CI $\:\left[.054,.177\right]$, and Dimension, $\:F\left(1,231\right)=40.35$, $\:p<.001$, $\:{\widehat{\eta\:}}_{G}^{2}=.046$, 90% CI $\:\left[.012,.098\right]$, and no significant interaction effect, $\:F\left(2,231\right)=1.06$, $\:p=.347$, $\:{\widehat{\eta\:}}_{G}^{2}=.003$, 90% CI $\:\left[.000,.016\right]$. Mandarin speakers on average underestimated the positions more strongly than English-speakers (mean difference of 1.22 units, *SE* = 0.23), *p* < .001, and German-speakers (mean difference of 1.37 units, *SE* = 0.24), *p* < .001 (cf. Figure 2). The difference between English and German-speakers was not significant (-0.15 units, *SE* = 0.23), *p* = .79. Also, the underestimation was on average stronger for the horizontal than vertical line orientation (mean difference of -0.75 units, *SE* = 0.12), *p* < .001. For the dependent variable *Absolute* positioning bias, however, there was only a significant main effect of Mother-tongue, $\:F\left(2,231\right)=27.66$, $\:p<.001$, $\:{\widehat{\eta\:}}_{G}^{2}=.168$, 90% CI $\:\left[.098,.238\right]$, but not of Dimension, $\:F\left(1,231\right)=0.79$, $\:p=.374$, $\:{\widehat{\eta\:}}_{G}^{2}=.001$, 90% CI $\:\left[.000,.015\right]$, and again no significant interaction, $\:F\left(2,231\right)=0.21$, $\:p=.812$, $\:{\widehat{\eta\:}}_{G}^{2}=.000$, 90% CI $\:\left[.000,.000\right]$. Mandarin speakers had a larger absolute positioning bias than English-speakers (mean difference of -0.94 units, *SE* = 0.18), *p* < .001, and German-speakers (mean difference of -1.35 units, *SE* = 0.19), *p* < .001. The difference between English and German-speakers was again not significant (0.40 units, *SE* = 0.18), *p* = .07.

### Response times

**Fig. 3:**
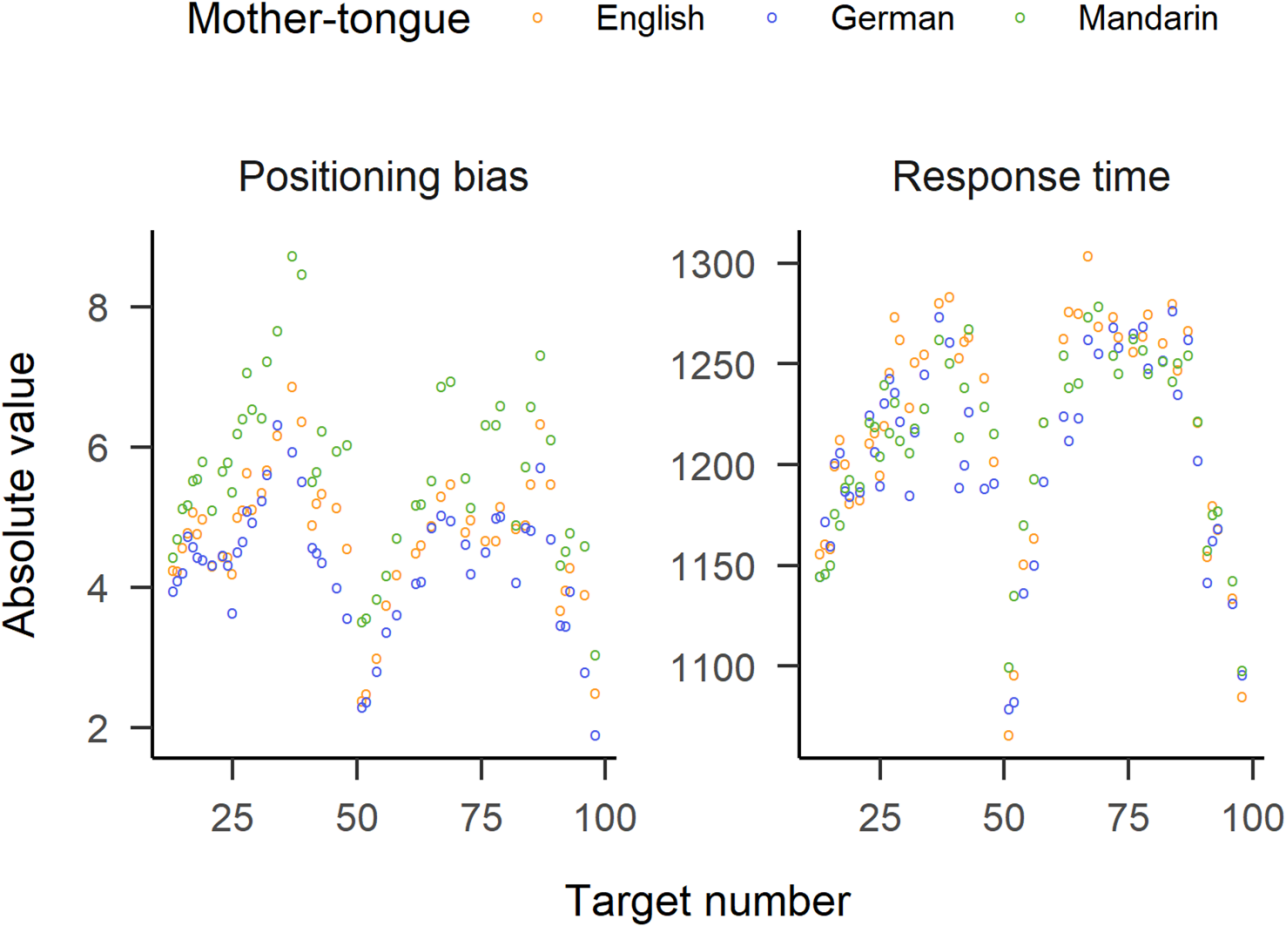
Absolute positioning bias (in target units, left panel) and response time (in ms, right panel) for the three language groups (colours, see legend). Note that the absolute value of the positioning bias is used here.

Figure 3 reflects the similar patterns of RT and absolute positioning bias in each language group. Visual inspection shows no speed-accuracy trade-off.

### Preregistered analyses

#### H1: Between languages comparison

There was no significant difference between native German- and English-speakers’ average distance between placing positions of inverted number pairs, $\:t\left(142.56\right)=0.08$, $\:p=.532$, Cohen’s *d* = 0.01. On average, German-speakers’ distance between placing positions of inverted number pairs was 28.41 units, *SD* = 1.79, and in English-speakers it was 28.38 units, *SD* = 2.52.

#### H2: Within English-speakers comparison

Native English-speakers on average underestimated both teen-numbers’ positions (mean positioning bias of -1.02 units, *SD* = 3.50), and twenty-numbers’ positions (mean positioning bias of -2.28 units, *SD* = 2.97). The difference between the average size of the positioning biases was significant, $\:t\left(79\right)=5.11$, $\:p<.001$, Cohen’s *d* = 0.57.

### Non-preregistered analyses

#### Teen- versus twenty-numbers in all languages

Regarding H2, even though the significant difference we hypothesized was present in the expected direction, we had postulated that numbers of the first decade should be overestimated instead of less underestimated. Both teen-numbers and twenty-numbers were underestimated with positioning biases significantly below 0; for teen-numbers $\:t\left(79\right)=-2.61$, $\:p=.011$, Cohen’s *d* = -0.29; for twenty-numbers $\:t\left(79\right)=-6.85$, $\:p<.001$, Cohen’s *d* = -0.77.

**Fig. 4:**
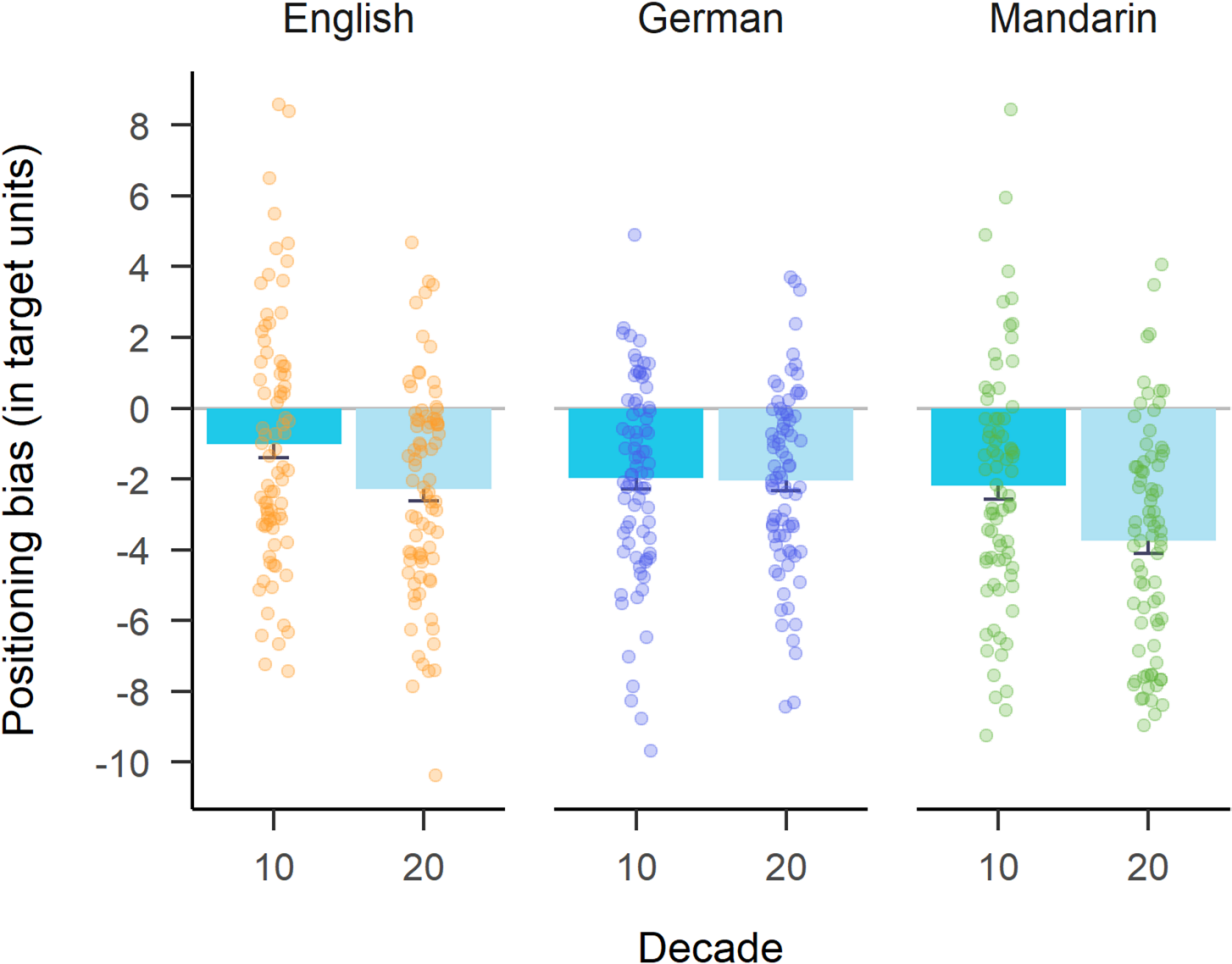
Mean positioning bias per decade (x-axis) and language group (panels). Points are individual participant data, i.e., mean positioning bias averaged over target numbers 13–19 and 23–29, respectively. Error bars display the standard error of the mean.

A paired *t*-test for German-speakers revealed no significant difference between the positioning biases from the two decades, $\:t\left(77\right)=0.23$, $\:p=.819$, Cohen’s *d* = 0.03. German-speakers significantly underestimated both teen-numbers (mean positioning bias of -1.97 units, *SD* = 2.79), $\:t\left(77\right)=-6.24$, $\:p<.001$, Cohen’s *d* = -0.71, and twenty-numbers (mean positioning bias of -2.04 units, *SD* = 2.57), $\:t\left(77\right)=-7.00$, $\:p<.001$, Cohen’s *d* = -0.79 (cf. Figure 4).

Executing the same analyses on data from Mandarin-speakers revealed that they, too, on average significantly underestimated both teen-numbers’ positions (mean positioning bias of -2.19 units, *SD* = 3.44), $\:t\left(75\right)=-5.54$, $\:p<.001$, Cohen’s *d* = -0.64, and twenty-numbers’ positions (mean positioning bias of -3.74 units, *SD* = 3.16), $\:t\left(75\right)=-10.30$, $\:p<.001$, Cohen’s *d* = -1.18. The difference between the average size of the positioning biases was significant, $\:t\left(75\right)=7.15$, $\:p<.001$, Cohen’s *d* = 0.82.

#### Intra-decade slopes

**Fig. 5:**
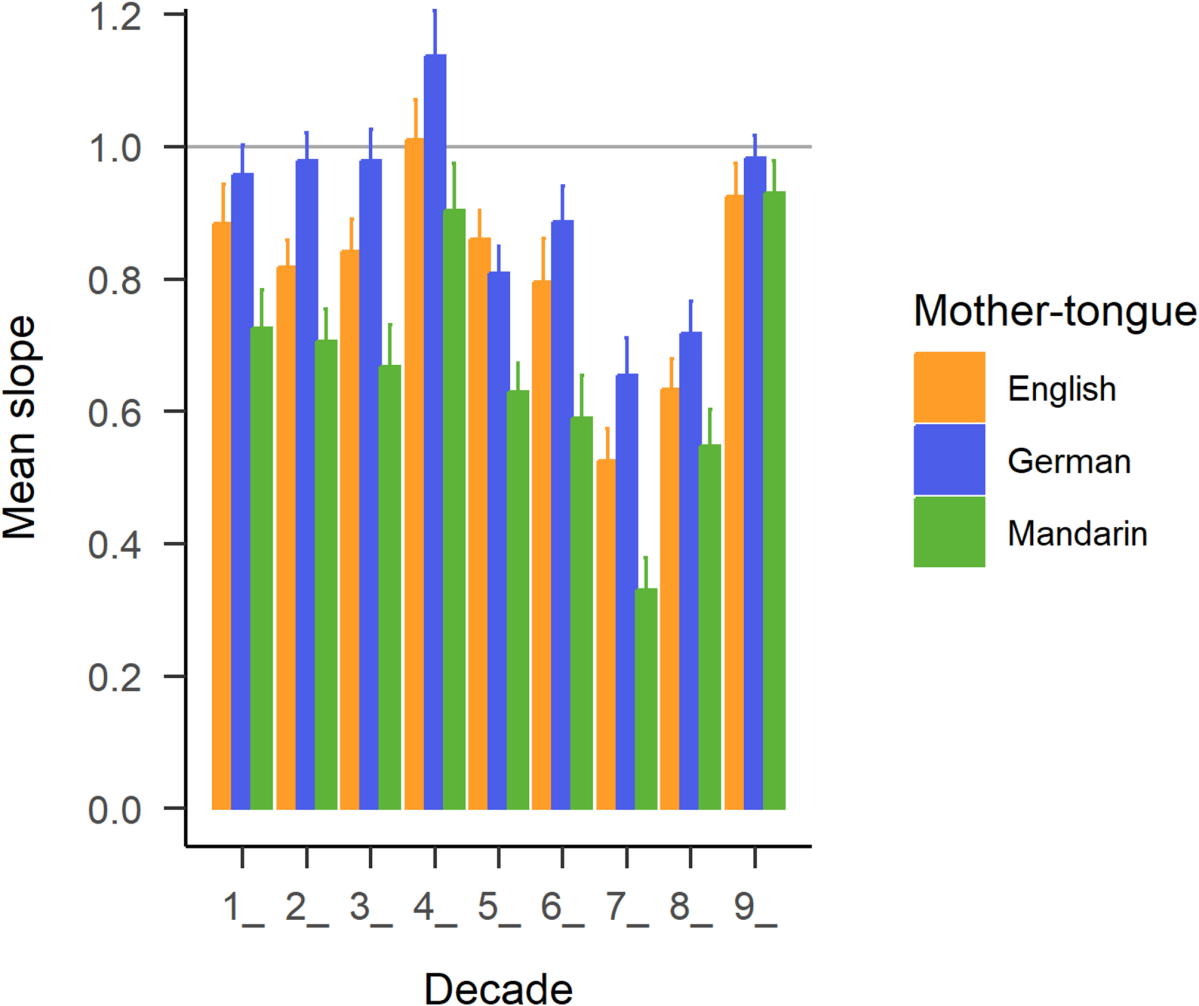
Mean slopes per decade (x-axis) and language group (colours, see legend). Horizontal grey line at y = 1.0 illustrates the hypothetical position of a ‘perfect’ slope for perfectly accurate responses. Note that the slopes for the first decade are only illlustrated here, but not used in the analyses, and that for analyses, slopes were averaged across decades. Error bars display the standard error of the mean.

German-speakers’ slopes were on average significantly larger (*M* = 0.89, *SD* = 0.24) than English-speakers’ slopes (*M* = 0.80, *SD* = 0.25), $\:t\left(155.99\right)=-2.37$, $\:p=.010$, Cohen’s *d* = -0.38, and than Mandarin speakers’ slopes (*M* = 0.66, *SD* = 0.28), $\:t\left(147.59\right)=-5.44$, $\:p<.001$, Cohen’s *d* = -0.88. English-speakers’ slopes were furthermore significantly larger than Mandarin speakers’, $\:t\left(149.17\right)=3.25$, $\:p=.001$), Cohen’s *d* = 0.52 (two-tailed), see Fig. 5.

#### Two-cycle power model with language modifier.

**Fig. 6:**
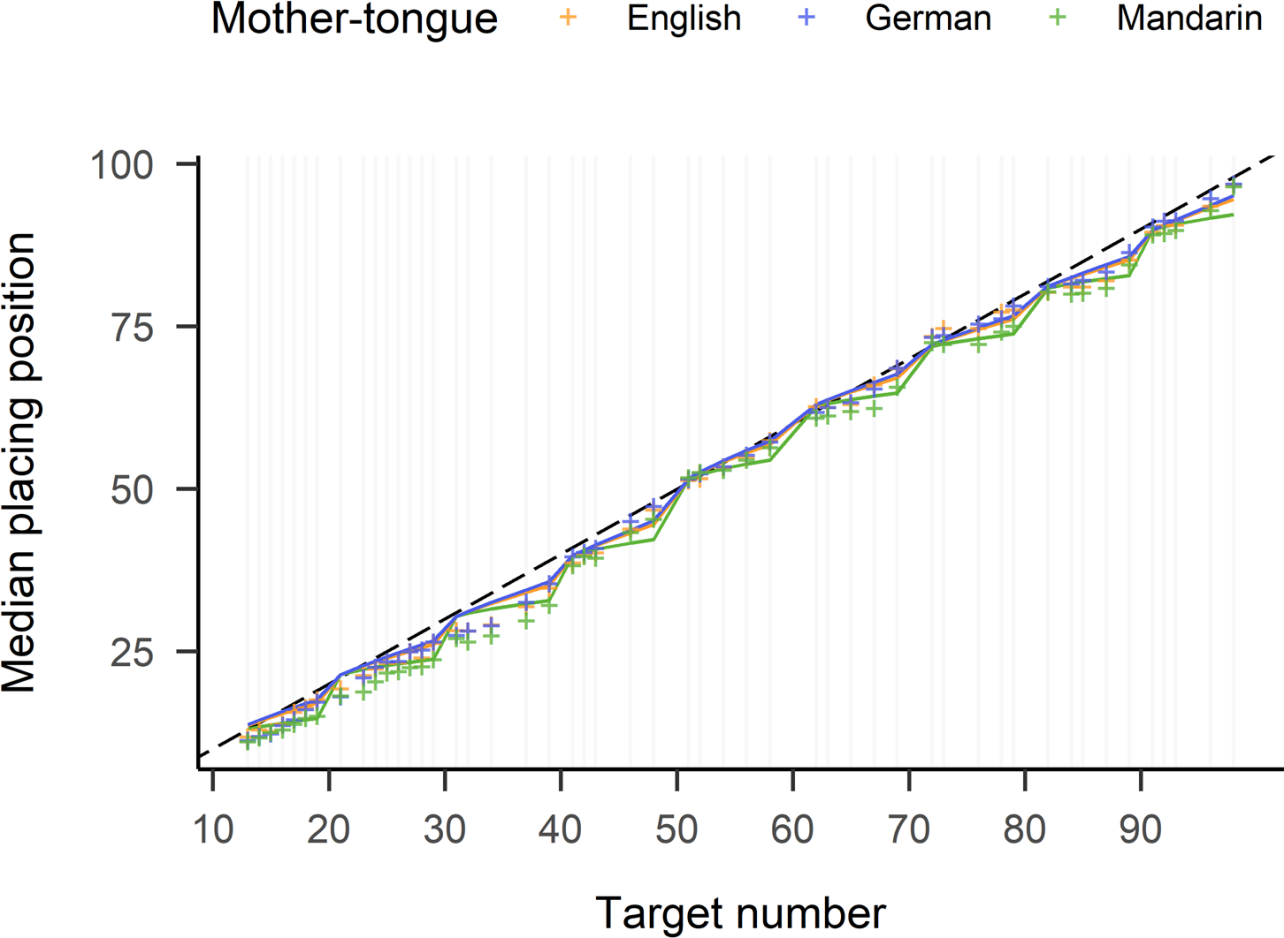
Median placing positions per target number for the three language groups (colours, see legend). The coloured lines represent modeled data. Vertical lines indicate target numbers used in the experiment. Note that the model was built on all target numbers > 20 and is applied also on smaller targets in this plot. The model deviates from the one reported in the text in that it was built with all three languages with English as the standard. See supplementary material for model specifications.

The three models described above were fitted to the data subsets. Table 2 shows all parameter estimates. It can be seen that the unit digits’ contribution was smallest in the Mandarin-speaking sample (see also Fig. 6). Within the fitted models, their $\:\delta\:$-value was significantly smaller by 0.24 than English-speakers’ $\:\delta\:$-value and by 0.28 than German-speakers’, *p*s < 0.001. German-speakers’ $\:\delta\:$-value was only nominally larger than English-speakers’ by merely 0.04, *p* = .27.

**Table 2:** Parameter estimates for the three fitted models.

Parameter	Estimate	Std. Error	t value	Pr(>|t|)
Model English - German				
β	0.892	0.018	49.713	0.000
δ	0.829	0.030	28.078	0.000
λ	0.044	0.040	1.112	0.269
Model English - Mandarin				
β	0.888	0.021	41.542	0.000
δ	0.831	0.035	23.477	0.000
λ	-0.239	0.066	-3.600	0.001
Model German - Mandarin				
β	0.908	0.022	41.639	0.000
δ	0.869	0.032	26.809	0.000
λ	-0.284	0.065	-4.374	0.000

Note. λ is the language modifier, modifying each model’s δ value for each secondly named language group in comparison to the standard, i.e. each first named language group.

## Discussion

This study was designed to investigate the effect of number word structure in different languages on number line estimation performance. To this end, languages with and without number word inversion were compared. The initially targeted two language groups - native English-speakers for a mostly non-inverting number system, and native German-speakers for an entirely inverting number system - were complemented by native Mandarin-speakers with an entirely non-inverting and highly regular number word system. In the German number system, in two-digit numbers, the unit digit is always spoken before the decade. In the English number system, this is only the case for teen-numbers. In the Mandarin number system, the decade is always pronounced before the unit digit. All language groups completed a non-verbal number line estimation (NLE) task, where, importantly, none of the target numbers was spoken aloud throughout the experiment session. This contrasts with other studies in which target numbers were presented orally^18^, where an effect would be attributable specifically to linguistic characteristics of number words. As expected, following previous findings, we found systematic deviations of number line estimations depending on the number word system also in the present non-verbal task. The analysis of linear regression slopes per decade appeared to be the most sensitive regarding the language-specific contribution of unit digits on placing positions. German-speakers had significantly steeper slopes than both English- and Mandarin-speakers, indicating relatively higher estimations of numbers with larger unit digits. This points towards the presence of an inversion effect, which had before only been shown in children^17^. Furthermore, the two-cycle power model with the newly introduced language modifier revealed language-specific influences, but did not detect a difference between English- and German-speakers. Instead, also German-speakers revealed a left-digit effect, as evidenced by a $\:\delta\:$ < 1, which reflects the larger contribution of the leftmost digit on placing positions^19,30–32^. Seemingly, the relatively larger contribution of the unit digit in this language group is not strong enough to override the very large contribution of the leftmost digit in determining the placement positions for two-digit numbers on a 0-100 number line. Moreover, the comparison of the average positioning bias of teen-numbers (13–19, inverted number words) with twenty-numbers (23–29, non-inverted number words) within English-speakers showed a stronger underestimation of twenty-numbers than of teen-numbers. Considering the general underestimation for almost all numbers, this could indicate a relative overestimation of teen-numbers. German-speakers (with inverted number words for both decades) revealed no significant difference between the positioning biases from the two decades.

Maybe the most striking finding regards the results patterns of Mandarin-speakers. In particular, they exhibited a much stronger left-digit effect than the two other language groups. Furthermore, the within-language comparison of teen- versus twenty-numbers yielded a response pattern similar to English-speakers, apart from the generally greater underestimation. Mandarin-speakers, just like English-speakers, showed a significantly less extensive underestimation for teen than twenty-numbers, although neither of those number words are inverted in Mandarin. Here, it is worth taking a closer look at the Mandarin number word system. Numbers one to ten have distinct number words. Numbers eleven to 19 are composed of the decade word for “ten” and the respective word for the unit digit (e.g., *13* is *“ten three”*). All further decades (i.e., 20, 30, 40, etc.) are composed of the word for the first digit and the multiplier “ten” (i.e., *“two ten”*, *“three ten”*, *“four ten”*, etc.), again followed by the respective word for the unit digit (e.g., *23* is *“two ten three”*). This is also how the Chinese script for these number words is composed. As a result, the leftmost digit, when written in Chinese script, or respectively the first spoken number word, is the largest for all teen-numbers, drastically shrinking when crossing the threshold into the twenty-numbers (i.e., 19–20 is “***ten*** *nine*”–“***two*** *ten*”). If one assumes that number names, even when not spoken out loud - influence their positioning, one should indeed assume that teen-numbers tend to be overestimated relative to their larger neighbouring decades.

### How does language influence placing positions in the NLE task?

The NLE task usually requires the translation of an Arabic number or a number word into a position on a line. There are at least two possibilities where language characteristics can exert influence on placing positions. First, they can already have biased specific mental number representations during a child’s learning process, e.g., for German-speaking children *29* may actually be a larger number than for English and (especially) Mandarin-speaking children (and stays so for adults). This corresponds to the (weak) hypothesis of linguistic relativity. Second, they can directly influence the translation process, e.g., thinking of the German *neunundzwanzig* (*“nine-and-twenty”*) may bias participants’ motor response towards the larger end of the number line. This can also happen in non-verbal tasks. Visual representation of numerals (Arabic numbers) and linguistic (verbal) representation of number words are two of the abstract symbolic codes that are part of a comprehensive representation of numbers, as postulated by current theories (Triple-Code Model^3^, sensorimotor perspective on numerical cognition^20)^. These symbolic codes co-activate each other whenever number symbols are processed, depending on the task more or less strongly. Therefore, the verbal code can influence responses also in a non-verbal task. This latter potential explanation does not relate to the linguistic relativity hypothesis. It could be true alternatively or additionally to the former potential explanation which would assume characteristics of a specific language to influence the mental representation of the referred non-linguistic concepts.

### The inversion effect

One interpretation of the inversion effect is that in languages with number word inversion, the unit digit contributes more strongly to mental number representation and, thus, then also to placing positions, because the unit is spoken first, whenever the whole two-digit number is pronounced. Accordingly, Helmreich and colleagues^17^ reported differential effects of mother-tongue on placing positions in Italian- and German-speaking (Austrian) children. Importantly, in their study, target numbers were only presented visually as Arabic numbers, indicating either a subvocalisation on the part of the participating children, or the automatic co-activation of the verbal code as described above. In our preregistered hypotheses, we had also focused on the inversion effect and chose to test it between languages with a direct comparison of the average distance in average placing positions between pairs of inverted numbers (e.g., *25* and *52*). However, this comparison was rendered difficult due to confounding factors and to a lack of sensitivity regarding the hypothesis of interest. The more sensitive analysis of linear regression slopes per decade indeed revealed German-speakers’ significantly steeper slopes than both English and Mandarin speakers’, indicating relatively higher estimations of numbers with larger unit digits. Effect sizes ranged from large for the comparison of German- and Mandarin-speakers’ slopes, to small for the comparison of German- and English-speakers’ slopes. The elusiveness of the inversion effect could imply that there is no, or only a very weak automatic co-activation of the verbal code in adults, or that the effect in previous studies instead arose from children (sub)vocalising the number words, which might happen to a lesser extent in adult participants. Future research should investigate whether the effect depends on subvocalization of the number words, e.g. by using a dual-task design involving articulatory suppression. Additionally, it might be the case that the effect is masked by another one with the opposite effect direction, e.g., the left-digit effect.

### The left-digit effect

The left-digit effect can, in the present context, be viewed as the *inverse* of the inversion effect in two-digit numbers: it reflects the larger contribution of the leftmost digit on placing positions^19,30–32^. Thus, in contrast to the inversion effect, it does not reflect a signature of number words, but of their visual representation as Arabic numbers, which are read from left to right. To investigate the left-digit effect, we used a two-cycle power model^19^ and introduced a new language modifier. The mother-tongue of the participants in the study by Patalano and colleagues^19^ is not reported, but the left-digit effect became evident in their model. We now showed that the left-digit effect is modulated by membership of a specific language group. Interestingly, also German-speakers revealed the left-digit effect, as evidenced by a $\:\delta\:$ < 1. In Mandarin-speakers, the left-digit effect furthermore seemed to be amplified by the specific structure of Mandarin number words. Accordingly, the decades’ relative contribution was significantly stronger not only than in German-speakers, but also than in English-speakers, who also do not have an inverting number word system.

,In other studies, the left-digit effect was investigated, instead of using a modeling approach, by assessing target pairs around decade breaks (e.g., 18/22, 29/31, etc., see e.g.^18,31,33,31,^^18,33^. The authors then analysed how much the distance between the numbers of the pairs’ placing positions exceeded their true numerical difference (“decade difference score”). Average decade difference scores above zero indicate a left-digit effect. Mirroring the analyses of Savelkouls and colleagues^18^ for the data of the present study (for detailed analyses, see supplementary material) not only closely replicates their results for the English monolingual groups, but also confirms the present study’s result patterns from the modeling approach. Where Savelkouls et al.^18^ find decade difference scores of *M* = 1.15, *SE* = 0.36 (when excluding the number pair 8/12 which contains a one-digit number), the present data yield a mean difference score of *M* = 1.19, *SE* = 0.21 for English-speakers, with a smaller, yet still significantly above zero, difference score of *M* = 0.82, *SE* = 0.20 for German-speakers, and by far the largest difference score of *M* = 2.34, *SE* = 0.23 for Mandarin-speakers.

### Interaction of the inversion effect and the left-digit effect

The left-digit effect in German-speakers was only descriptively weaker than in English-speakers, where we might have expected the effect to be more strongly attenuated in German-speakers due to their assumed focus on the unit digit. In fact, Savelkouls et al.^18^ did report an absence of a left-digit effect in a group with an inverting mother-tongue. In addition to English monolinguals (*N* = 20), they tested Dutch-English bilinguals (*N* = 40) in a verbal NLE task, where target numbers were read out aloud by the experimenter. The monolingual group showed the expected left-digit effect. Interestingly, the left-digit effect was absent in the bilingual group not only when numbers were read in Dutch (a language with number word inversion), but even when the numbers were read in English. The seemingly contradictory findings can be reconciled if one assumes different origins of the effects. The left-digit effect seems to depend more strongly on the presentation format than the inversion effect, possibly resulting from the visual features inherent in two-digit numbers’ place-value structure. That is, the effect might especially occur for the visual representational code of numbers, because of the left-to-right reading direction and the overlearnt fact that the left digit encodes the objectively more influential information regarding the number’s size. The inversion effect, on the other hand, might arise at least partly from persistent (biased) mental number representations. These may form when children learn and consolidate the meaning of numbers in a specific language with all its characteristics. In the study by Savelkouls et al.^18^, the inversion effect overrode any potentially existent left-digit effect in Dutch-speakers, which did not happen in the present study’s German-speakers, possibly because the visual (as opposed to verbal) presentation of target numbers promoted the left-digit effect.

### General result patterns and language-specific estimation performance

The response time and positioning data from the tablet task (as well as positioning data from the floor task, see supplementary materials) show expectable global patterns in participants’ number line estimation performance. The middle and the ends of the line serve as anchors to more proficiently estimate target numbers close to 50 and 100 (target numbers close to 0 were not used). Numbers farther away from these anchors were on average responded to more slowly and less accurately (see Fig. 3). However, on top of these global patterns, we found that the investigated language groups differed in specific ways. The originally assumed number word inversion effect in German-speakers revealed to be much smaller than expected, but detectable. The largest effect seems to have been exerted by the Mandarin multiplier-based number system, not only amplifying the left-digit effect, but at the same time decreasing their average estimations for almost all of the target numbers, consequently leading to a less accurate estimation performance. We could not find reports of NLE tasks performed by Chinese adults that would allow for a comparison of the systematic underestimation found in the current sample. Moreover, in the literature, mostly the PAE, thus using absolute error, is reported as a measure for accuracy in the NLE task, complicating such a comparison also for child participants. Still, given previous findings that performance in the NLE task correlates with mathematical competence^8–10^, and that Chinese participants outperform participants from Western cultures in mathematical tasks^9,34–39^, Chinese worse performance in the NLE task here comes as a surprise. However, the vast majority of studies on the relationship between performance in the NLE task and mathematical proficiency tested children. To the best of our knowledge, there is little to no evidence that such a significant correlation persists into adulthood (with the highest sample mean age of 14 years^8)^. A replication of Mandarin-speakers’ considerable underestimation in NLE, and an investigation of whether the correlation between NLE performance and mathematical performance extends cross-culturally into adulthood, remain issues for future research.

## Conclusion

The current study showed that membership of a specific language group modulates the left-digit effect. Our interpretation of the results is that not only did the German language’s number word inversion property (descriptively) attenuate the effect, but that the specific structure of Mandarin number words furthermore amplified it, in comparison to English-speakers. An inversion effect became evident in German-speakers in direct comparison with English-speakers. German-speakers displayed relatively higher estimations of numbers with larger unit digits, as indicated by significantly steeper linear regression slopes per decade than both English- and Mandarin-speakers. Overall, the study suggests that the cognitive processing of numbers is a complex phenomenon that is influenced by situational factors, such as presentation format, and that is persistently shaped by language, even in adults.

## Data Availability

The data, analysis script, supplementary materials, as well as the preregistration, are available under https://osf.io/nt9zv.
